# Engineering tyrosine residues into hemoglobin enhances heme reduction, decreases oxidative stress and increases vascular retention of a hemoglobin based blood substitute

**DOI:** 10.1016/j.freeradbiomed.2018.12.030

**Published:** 2019-04

**Authors:** Chris E. Cooper, Gary G.A. Silkstone, Michelle Simons, Badri Rajagopal, Natalie Syrett, Thoufieq Shaik, Svetlana Gretton, Elizabeth Welbourn, Leif Bülow, Nélida Leiva Eriksson, Luca Ronda, Andrea Mozzarelli, Andras Eke, Domokos Mathe, Brandon J. Reeder

**Affiliations:** a School of Biological Sciences, University of Essex, Wivenhoe Park, Colchester, Essex CO4 3SQ, United Kingdom; b Pure and Applied Biochemistry, Department of Chemistry, Lund University, Box 124, 221 00 Lund, Sweden; c Department of Medicine and Surgery, University of Parma, Parma, Italy; d Department of Food and Drug, University of Parma, Parma, Italy; e Institute of Biophysics, National Research Council (CNR), Pisa, Italy; f Department of Physiology, Semmelweis University, Budapest, Hungary; g Department of Biophysics and Radiation Biology, Semmelweis University, Budapest, Hungary

**Keywords:** Hb, Hemoglobin, Mb, Myoglobin, HBOC, Hemoglobin Based Oxygen Carrier, WT, wild type recombinant protein, metHb, met(ferric) hemoglobin, metMb, met(ferric) myoglobin, oxyHb, oxygenated hemoglobin, HbCO, carbon monoxide bound Hb, CP20, deferiprone, 1,2-dimethyl-3- hydroxypyrid-4-one, HEK, Human Embryonic Kidney cells, Hemoglobin, Oxidative stress, Blood substitute, Electron transfer, HBOC, PEGylation

## Abstract

Hemoglobin (Hb)-based oxygen carriers (HBOC) are modified extracellular proteins, designed to replace or augment the oxygen-carrying capacity of erythrocytes. However, clinical results have generally been disappointing due to adverse side effects, in part linked to the intrinsic oxidative toxicity of Hb. Previously a redox-active tyrosine residue was engineered into the Hb β subunit (βF41Y) to facilitate electron transfer between endogenous antioxidants such as ascorbate and the oxidative ferryl heme species, converting the highly oxidizing ferryl species into the less reactive ferric (met) form. We inserted different single tyrosine mutations into the α and β subunits of Hb to determine if this effect of βF41Y was unique. Every mutation that was inserted within electron transfer range of the protein surface and the heme increased the rate of ferryl reduction. However, surprisingly, three of the mutations (βT84Y, αL91Y and βF85Y) also increased the rate of ascorbate reduction of ferric(met) Hb to ferrous(oxy) Hb. The rate enhancement was most evident at ascorbate concentrations equivalent to that found in plasma (< 100 μM), suggesting that it might be of benefit in decreasing oxidative stress in vivo. The most promising mutant (βT84Y) was stable with no increase in autoxidation or heme loss. A decrease in membrane damage following Hb addition to HEK cells correlated with the ability of βT84Y to maintain the protein in its oxygenated form. When PEGylated and injected into mice, βT84Y was shown to have an increased vascular half time compared to wild type PEGylated Hb. βT84Y represents a new class of mutations with the ability to enhance reduction of both ferryl and ferric Hb, and thus has potential to decrease adverse side effects as one component of a final HBOC product.

## Highlights

• Cell free Hemoglobin Based Oxygen Carriers can deliver oxygen to tissue.
• Oxidation of ferrous hemoglobin to ferric and ferryl leads to oxidative toxicity.
• Insertion of tyrosine residues enhances reduction of ferryl to ferric hemoglobin.
• Some mutants (e.g. βT84Y) also enhance ferric to ferrous reduction by ascorbate.
• βT84Y decreases cellular oxidative stress and enhances vascular retention in vivo.

## Graphical abstract

**Figure:**
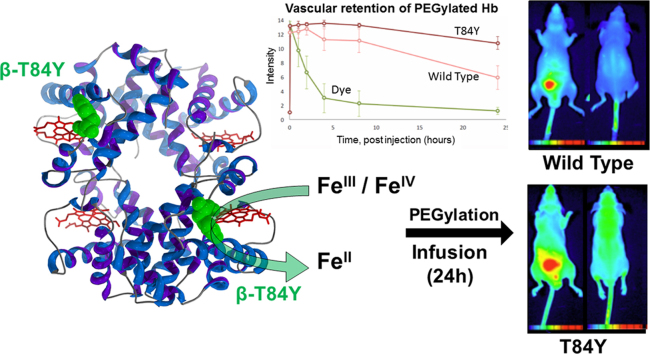

## 1 Introduction

Hemoglobin (Hb) Based Oxygen Carriers (HBOC, colloquially termed “blood substitutes”) have the potential to be transfused at high dose in place of packed red blood cells to restore impaired oxygen transport [1]. However, at lower doses HBOC products can be targeted not as a red blood cell replacement, but as a product that can deliver oxygen more efficiently to sites the red cell cannot reach, either due to abnormalities of red cell deformability (e.g. sickle cell crisis), regional tissue hypoxia (e.g. subarachnoid haemorrhage, stroke) or a disordered microvascular circulation that the red cell cannot traverse (as seen in trauma, sepsis and other acute inflammatory states). When used in this way as an “oxygen therapeutic”, HBOC products do not deliver bulk oxygen, but instead facilitate oxygen transfer from the red cell. In effect a high oxygen affinity HBOC is performing between the vasculature and the hypoxic tissue, the same role that myoglobin (Mb) plays within the muscle cell [2], [3] in enhancing oxygen diffusion.

Whatever their proposed in vivo role, HBOCs have historically faced issues in development due to adverse side effects caused by Hb toxicity. Outside the red blood cell, Hb toxicity is in part due to nitric oxide scavenging reactions [4], although it is possible to engineer decreased nitric oxide scavenging properties, thereby decreasing the vasoconstriction caused by extracellular Hb [5]. However, extracellular Hb also has an intrinsic oxidative reactivity that damages cells, proteins, lipids and DNA [6]. Additionally, free heme released from Hb acts as a Damage Associated Molecule Pattern protein (DAMP) molecule which can activate the immune system [7].

Maintaining Hb in its functional oxygen carrying ferrous state is key to its function as an HBOC. Most HBOC as delivered contain a fraction (≅5%) of Hb in the ferric state (metHb). The steady state level of methemoglobin is a function of the balance between its formation via the autoxidation of oxyhemoglobin and the reduction of methemoglobin via methemoglobin reductase systems. Inside the red blood cell the enzyme methemoglobin reductase converts methemoglobin to oxyhemoglobin using the reducing power of NADH, although non-enzymatic mechanisms can also contribute [8]. However, in the plasma this enzyme is not present. The reduction of HBOC is therefore powered by plasma antioxidants, in particular ascorbate [9] Some regeneration of the oxidized ascorbate is possible from intra-erythrocyte NADH via a trans-plasma membrane reductase system. However, plasma ascorbate levels are likely to be compromised in severe trauma and/or in humans and animals such as guinea pigs that have lost the ability to synthesize ascorbate. As a consequence, the ability to maintain the hemoglobin in an HBOC in its functional oxygen carrying ferrous form varies with both the HBOC itself, the animal model used and the level of trauma. Methemoglobin levels can therefore drop [10], stay the same [11], or increase [9] following HBOC addition.

The metHb levels are also important as increasing metHb induces oxidative stress in vivo by reacting with peroxides [12]. In the ferrous state this creates ferryl iron; in the ferric state, both ferryl iron and globin radicals are produced. Following peroxide addition to ferric globins, ascorbate can quantitatively capture both the ferryl iron and the free radical species [13]. However, kinetic limitations may result in partial reduction and an increase in tissue oxidative damage. Using recombinant tools it is possible to design a protein to be less oxidatively reactive [14]. Tyrosine amino acids are able to act as redox mediators by cycling between oxidized (radical) and reduced forms. Mb [15] and the Hb α-subunit, but not the β-subunit [16] have electron transfer pathways that are able to enhance the rate of ferryl reduction by plasma antioxidants. These proteins have a high affinity saturable pathway. Introducing such a pathway in the β-subunit in the homologous site where one is present in the α-subunit (βF41Y) resulted in enhanced ferryl reduction in tetrameric hemoglobin [16]. A range of mutations was then introduced into a model system – Mb from the Sea Hare (*Aplysia fasciata*), which lacks any tyrosine residues. A variety of phenylalanine to tyrosine mutations were engineered with enhanced electron transfer pathways for ascorbate [17]. In the presence of ascorbate, but not in its absence, these mutants had significantly decreased lipid peroxidation activity compared to the wild type (WT) protein. A similar effect was seen in the human βF41Y mutation. However, the rate constant enhancement seen in the *Aplysia* mutations was orders of magnitude greater than that seen following the human mutation. To that end we engineered a range of new tyrosine mutations in the human Hb α and β subunits to see if a similar large increase could be observed. Surprisingly, we found that a subset of these mutations were also able to facilitate the reduction of ferric iron to ferrous iron i.e. return ferryl Hb to a fully functioning ferrous oxy/deoxy oxygen transport molecule. As the ferric form of Hb is the most unstable, triggering heme loss and protein degradation [18], we tested in vivo the mutant best able to reduce ferric to ferrous (βT84Y) to see if these properties, once incorporated into a functioning HBOC, also enhanced vascular half-time.

## 2 Experimental

### 2.1 Protein preparation

The cloning, expression and purification of the Hb proteins for in vitro studies was carried out as previously described [16] and the proteins stored in the stable ferrous CO adduct form (HbCO). The conversion of the stored ferrous CO adduct to the ferric (met), ferrous (deoxy) and ferrous (oxy) were as in [19]. For enhanced purification for cellular studies additional anion exchange and metal affinity steps were introduced [20]. Scale up from shake flasks to fermentation growth was required to produce enough protein for the animal studies. BL21-DE3 *E. coli* cells carrying the adult or fetal Hb gene were cultivated in a 5 L fermenter (BioStat A, Sartorius). Hb was grown according to methods previously described [21], [22] with minor modifications (glucose 70% was used as carbon source and no acid feed was used). The production of Hb was induced by adding IPTG (0.5 mM) and δ-aminolevulinic acid (2 mM). Twelve hours after induction, the cells were harvested by centrifugation. The harvested cells were lysed in buffer, Tris-HCl 50 mM pH 8.5. Hb was purified using two-step ion exchange chromatography, first a cation (Capto S) and then an anion (QHP) exchange. Prior to the first step, the lysis buffer was exchanged to buffer sodium phosphate (NaPi) 10 mM pH 6.0. Hb was eluted in one step with NaPi 70 mM pH 7.2. For the second step, the buffer was again exchanged to buffer Tris-HCl 20 mM pH 8.3. Hb was eluted with 10 column volume linear gradient of NaCl from 0 to 100 mM in NaPi 50 mM pH 6.2. Just before and after every purification step, the solutions of Hb were bubbled with carbon monoxide (CO) to keep it in the oxidatively inert CO-ligated form. The protein was then stored as the ferrous CO adduct (HbCO) at − 80 °C.

### 2.2 Preparation of PEGylated hemoglobin

CO removal from stored Hb was carried out by illuminating CO hemoglobin samples through a 75 W Xenon to promote photolysis under 100% oxygen flow. CO removal and substitution with oxygen was confirmed by collecting absorption spectra. All concentrations of Hb used were calculated as the tetrameric form, and were commonly 60 µM. Inositol hexabisphosphate (IHP) was added to the Hb solutions to give the ratio of 1.2:1 (tetramer) and deoxygenated under helium flow and gentle shaking for 4 h, at 20 °C. Iminothiolane (IMT) was added to the Hb solutions to give the ratio of 80:1 (IMT:tetrameric Hb) and after 6 min maleimido-PEG added to give the ratio 12:1 (PEG:Hb tetramer). The reactions was stopped with 5.3 M lysine pH 6.5–7.5 after 34 min and 0.9 M cysteine after 46 min. All solutions were prepared using deoxygenated water and kept under nitrogen flow before addition. PEG-Hb solutions were dialyzed in phosphate buffered saline (PBS) using Spectrum Hollow Fibers (50,000 MW cut-off) to remove unreacted PEG, IMT, lysine, cysteine and IHP. Endotoxin was removed as described previously [23]. The level of endotoxin of the injected samples was 34 EU/mL for the wild type and 11 EU/mL for βT84Y. PEGylated Hb (PEG-Hb) samples were aliquoted into separate 5 mL vials, flash frozen in liquid nitrogen and stored at − 80 °C. prior to shipping for animal studies. A new vial was separately thawed for each animal to avoid protein degradation due to multiple freeze-thaw cycles.

### 2.3 Oxygen affinity measurements

Upon CO removal Hb solutions left overnight under helium flow in the presence of the Hayashi reducing system before titrations [24]. The oxygen p50 and Hill coefficients were obtained by measuring optical spectra after equilibration at varying pO_2_ gas mixtures as previously described [25]. p50 was measured in 100 mM Hepes, 1 mM EDTA, 100 mM sodium chloride, 1.2 sodium phosphate, pH 7.0, 25 °C. The fractional saturation was calculated fitting the data to a linear combination of oxy, deoxy and met Hb reference spectra.

### 2.4 Optical spectroscopy

Spectra were recorded on a Varian Cary 5E spectrophotometer. The autoxidation (k_obs_ min^-1^) of the rHb proteins was measured at 37 °C. For each protein, the autoxidation (oxy to met conversion) was measured, and the kinetic traces (577–630 nm) were analysed by single exponential fittings to give k_obs_ (min^-1^) values. Ferric reduction by ascorbate (met to oxy conversion) was measured at 25 °C in 20 mM NaPi, pH 7.20 and the extent (%) of oxyHb formed calculated with reference to the 100% met and oxyHb spectra of the relevant mutant. Heme loss from metHb (1.7 μM) was measured in 20 mM NaPi, pH 7.20, 37 °C by monitoring the high to low spin state change when heme binds to 2 µM of the high affinity heme scavenger hemopexin (Sigma, Aldrich). Spectra were taken between 370 and 650 nm and the 417–599 nm difference fitted to a single exponential decay. Ferryl hemoglobin was made by adding a 3:1 ratio of H_2_O_2_:metHb and waiting 10–15 min until the spectral change was complete (confirmed by the optical spectra). Trace catalase (1–5 nM) was then added to remove unreacted H_2_O_2_. Ferryl reduction (ferryl to met) by the reductant CP20 (deferiprone, 1,2-dimethyl-3-hydroxypyrid-4-one) was analysed by double exponential fits of the time courses (545–630 nm), assigning the faster phase to the α subunit and the slow phase to the β subunit as described previously [16]. The autoreduction rate constant at zero [CP20] was then subtracted from the rate and the corrected data fit to Michaelis-Menton kinetics using non linear regression (errors are SEM from the curve fits). The SEM of the ratio V_max_ /K_m_ was approximated from the respective SEM assuming the variables were independent and using a first order Taylor expansion. Var(x/y) = x^2^/y^2^ * ( Var(x)/x^2^ + Var(y)/y^2^).

### 2.5 Cell assays

The metHb proteins were prepared as previously stated [19]. OxyHb proteins were prepared by incubating HbCO under bright constant illumination whilst gently blowing a stream of pure oxygen over the surface of the liquid, and maintained on ice. Conversion to the oxyHb form was monitored spectrally and was complete in about 30–45 min. Human Embryonic Kidney cells (HEK), was obtained from Lonza. Cells were maintained in complete DMEM media (Lonza) in a humidified atmosphere with 5% CO_2_ at 37 °C until confluent. For experiments to test the effect of rHb on cells, cells were trypsinized, counted, plated into 24 well plates and maintained until 70% confluent. Prior to all treatments, cells were gently washed with warm phosphate buffered saline and the media was replaced by serum and phenol free DMEM. Cells were treated by adding 50 μM Hb (oxy or met), with or without 50 μM ascorbate to the media to a total volume of 250 μL. Cells were placed a plate-reader (Tecan Infinite M200Pro) at 37 °C, 5% CO_2_ and absorbance spectra 500–650 nm collected every 10 min for 18 h. Kinetic traces were produced by a three-point drop correction across the 577 nm oxy peak using 562 and 592 nm as reference wavelengths. Cellular damage was assessed by measuring levels of lactate dehydrogenase (LDH) released into cellular media after 18 h of exposure to the experimental treatment. The LDH assay (Sigma, Aldrich) was performed according to manufacturer's instructions. The absorbance at two wavelengths (450 and 650 nm) was obtained using a Tecan Infinite M200Pro plate reader and both values were used to calculate levels of LDH.

### 2.6 Animal studies

Following endotoxin removal, the PEG-Hb was lablelled with a Cyanine5 dye NHS ester (Cy5-NHS; Cy5 for short) red fluorescent dye (Lumiprobe, Germany). The pH of the PEG-Hb solution was adjusted to 8.5 with 400 μL of 0.1 M NaHCO_3_ in an aliquot of 0.8 mL. Next, 1 mg Cy5 was dissolved in 200 μL dimethyl sulfoxide (DMSO, Sigma-Aldrich, Germany). Subsequently, 1000 μL of PEG-Hb solution and 200 μL Cy5 solution was stirred and mixed at 400 rpm for 5 h at room temperature. The resultant reaction mixture was purified to remove unreacted Cy5 using Size Exclusion Chromatography (SEC) in a NAP 10 column (GE Life Sciences, Marlborough, MA, USA) for cleaning in two steps (1000 μL and 200 μL, each) using the eluent physiological saline and was collected as in a volume of 1200 μL. For further purification details and characterization see Supplementary Fig. S1.

Animals were obtained from Charles River GmbH, Germany. NU(NCr)-Foxn1nu/nu nude mice (n = 5 per group, mixed gender, body weight of 23–33 g) were allowed free access to food and water and maintained under temperature, humidity, and light-controlled conditions. All procedures were conducted in accordance with the ARRIVE guidelines and the guidelines set forth by the European Communities Council Directive (86/609 EEC) and approved by the Animal Care and Use Committee of the IEM and the Semmelweis University (XIV-I-001/29–7/2012). In the fluorescent whole-body imaging experiments, the animal was initially anesthetized by Medetomidine (1 mg/kgbw) + Ketamine (100 mg/kgbdw). Anesthesia was maintained using continuous inhalation of 2% isofluorane in medical grade oxygen gas. While being scanned, the animal was placed on heating mat to maintain the rectal temperature at 37.5 °C.

The whole-body biodistribution of Cy5-labeled PEG-Hb was measured in vivo at pre-injection and at different post-injection time points (0, 1, 2, 4, 8, 24 h). Anesthetised mice were infused by top-loading via the tail vein at a rate of 0.2 mL/min [4]. Animals involved in these studies showed no signs of any manifest cardio-respiratory distress. Sample selection (control, αV1M βV1M or test αV1M βV1M βT84Y) was randomised and blind to those doing the study and analysis. Before scanning, 0.1 mL 1 mg/mL Cy5 dissolved in saline was administered to control mice via the tail vein, while treated animals got the same amount of Cy5 dye in the form of Cy5-labeled PEG-Hb dissolved in saline; the PEG-Hb used was 2.4 mM heme (3.84%) for αV1M βV1M and 2.2 mM (3.52%) for αV1M βV1M βT84Y. The animals were scanned in two dedicated (prone and supine) positions with the combination of a 630 nm light source and a 725 nm long-pass emission filter for detection using a FOBI™ whole-body imager (Neoscience Ltd., Suwon City, Rep. Korea) at 1392 × 1040 pixel detector and 24 bit intensity resolution. The exposure time was 1000 ms per image with gain level 1. Prior to fluorescence scanning, background images were registered in the same position using white light illumination (400–800 nm) without applying any emission filter for detection. In these cases, the exposure time was 200 ms with gain level 1.

The blood level of labeled PEG-Hb and free Cy5 was measured by smearing one drop of 100 μL blood freshly drawn the from the tail vein onto the white plastic surface of a standardized balance plastic sample holder (4 cm^2^). After a calibration measurement of the sample holder's autofluorescence, the same holder with the thin blood film was put into the FOBI optical imaging system and imaged with integration time of 1000 ms and gain level 1. This procedure allowed for repetitive measurements of blood levels of Lumiprobe Cy5 dye either in its free form (control) or bound to PEG-Hb throughout a period of 24 h. Lumiprobe Cy5 NIR fluorescence intensity was normalized to the pre-injection level (i.e. autofluorescence). Following the last imaging time point (24 h) whole-body saline perfusion was applied for 6 min in order to clear blood from the organ tissues. Subsequently, organs were removed and their ex vivo fluorescent images taken with 1000 ms exposure and gain level 1. Mice were terminally anesthetised by the dedicated veterinary use euthanasia drug Euthasol^®^ (ad us. vet. injection).

## 3 Results

**Fig. 1:**
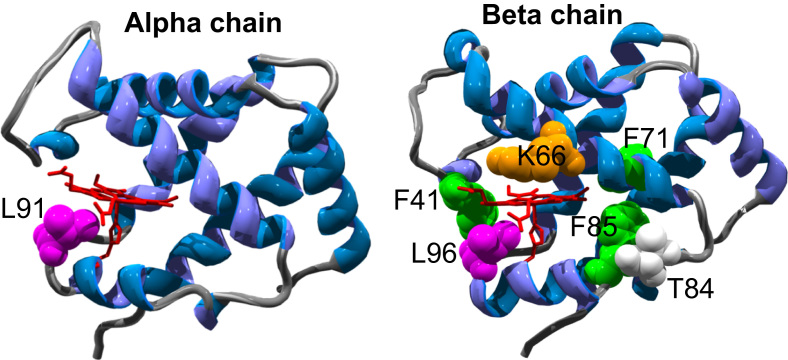
**The three dimensional α- and β-chain structures of HbA.** Illustrating the position of the novel mutations introduced. Structure file 2DN2 from the Protein Data Bank (www.pdb.org/pdb) was used to generate the figure. Heme and proximal histidine is in red (stick), sites of mutations are in green, orange, pink or white (spacefill).

Fig. 1 illustrates the location of the novel mutations introduced into the human Hb α and β subunits. Table 1 describes the rationale for the design of each mutation. All the engineered mutations could readily be grown in shake flasks, purified and converted into the met, deoxy, oxy and CO forms. In general (Table 2), introducing tyrosine residues led to only minor (1–2 nm) differences from the optical absorbance peaks seen in WT Hb. Some larger changes were seen in the visible region in the metHb spectra. This is not unexpected as these are broad peaks and sensitive to the heme iron spin state, which is pH dependent; a small change in the pK of the ferric heme would therefore lead to minor changes in peak position. The one notable exception is βK66Y, which does show larger changes in the deoxy and (especially) met forms. In particular the significant red shift in the Soret peak (407–413 nm) suggests that a large fraction of the ferric heme in the met form is low spin at neutral pH, given that low spin complexes have red shifted Soret peaks e.g. cyanide at 419 nm and azide at 417 nm [26]. This was confirmed by low temperature EPR spectroscopy which showed a large fall in the *g* = 6 high spin metHb signal in βK66Y compared to WT. All mutants were able to reversibly bind oxygen, although the binding affinity differed from WT in some cases. The most striking difference was the high affinity of the βK66Y mutant and the low affinity of αL91Y and βL91E/βL96Y.

**Table 1:** Rationale for design of mutation.

**Mutation**	**Rationale for design**
WT	–
βF41Y	Equivalent to Y42 in α subunit of Hb and F42Y in Aplysia (Mb); both natural (α) and introduced (*Aplysia*) mutations have been shown to enhance electron transfer rates from external reductants to ferryl heme [15], [16], [17]
βK66Y	A new surface exposed Tyrosine introduced on the distal side of heme pocket (all others are on the proximal side)
βF71Y	A new Tyrosine introduced at the back of the heme pocket that is not surface exposed but within electron transfer distance of the surface (a control as to whether surface exposure is necessary for enhanced reduction of ferryl heme).
βT84Y	A new Tyrosine introduced at the back of the heme pocket that is surface exposed
βF85Y	A new Tyrosine introduced at the back of the heme pocket that is surface exposed, but with a longer distance to heme edge (~ 10 Å)
αL91Y	Position equivalent to F98Y in *Aplysia* Mb (a mutation that showed much faster electron transfer to ferryl heme even than that seen in WT α Hb [17]). An attempt to see if the already fast reduction of ferryl heme in α Hb can be accelerated.
βL96Y	Equivalent to F98Y in *Aplysia* Mb but now positioned with respect to β heme subunit.
βL91E/βL96Y	The addition of L91E is in a position to stabilize L96Y deprotonation through H-bonding and hence accelerate the ferryl heme reduction rate (forming a similar role to that attributed to E94 in *Aplysia*[17]).
αL91Y/βT84Y	Combining mutations to test for a synergy of effects on ferric reducibility
αV1M βV1M	Prevent extended N terminal length due to deficiency of methionine cleavage in *E.coli*. A standard tool used in preparing recombinant Hb for large scale production by fermentation required for animal trials [22].
αV1M/βV1M βT85Y	Combining mutations to enable test of enhanced reducibility in animal models

**Table 2:** **Optical and oxygen binding properties of mutant proteins.** The wavelength peaks of the optical bands in the visible (alpha/beta peaks) and Soret (gamma peak) for the met, deoxy, oxy, and carbonmonoxy species following the introduction of new tyrosine residues into HbA. Conditions: sodium phosphate (20 mM, pH 7.20); T = 25 °C; [heme] = 10 μM. Oxygen affinities were measured at 100 mM HEPES, 100 mM sodium chloride, 1.2 mM sodium phosphate, 1 mM EDTA, pH 7.0, T = 25 °C.

**Mutant**	WT	βF41Y	βK66Y	βF71Y	βT84Y	βF85Y	αL91Y	βL96Y	βL91E/βL96Y
**MetHb**									
alpha	*577*	*579*	*567*	*579*	*579*	*578*	*581*	*573*	*583*
beta	*540*	*541*	*537*	*541*	*542*	*542*	*536*	*540*	*542*
gamma	*407*	*407*	*413*	*407*	*407*	*407*	*407*	*407*	*407.5*
**DeoxyHb**									
alpha	*582*	*584*	*587*	*584*	*583*	*583*	*583*	*584*	*584*
beta	*557*	*556*	*560*	*557*	*553*	*556*	*556*	*557*	*557*
gamma	*431*	*431*	*429*	*431*	*431*	*431*	*431*	*431*	*431*
**OxyHb**									
alpha	*577*	*577*	*577*	*577*	*577*	*577*	*577*	*577.5*	*577.5*
beta	*542.5*	*543*	*543*	*543*	*543*	*543.5*	*544*	*543.5*	*543.5*
gamma	*417*	*417*	*417*	*417*	*417*	*416.5*	*417*	*417*	*416.5*
**HbCO**									
alpha	*570*	*570*	*571*	*570*	*570*	*571*	*572*	*571*	*571*
beta	*541*	*541*	*541*	*540*	*539*	*541*	*542*	*540*	*542*
gamma	*419*	*419*	*421*	*419*	*420*	*419*	*420*	*420*	*420*
**p50 (torr)**	5.21 ± 0.07	8.89 ± 0.51	1.17 ± 0.10	2.82 ± 0.37	7.16 ± 0.37	5.12 ± 0.17	14.91 ± 0.60	3.67 ± 0.60	9.63 ± 0.35

**Fig. 2:**
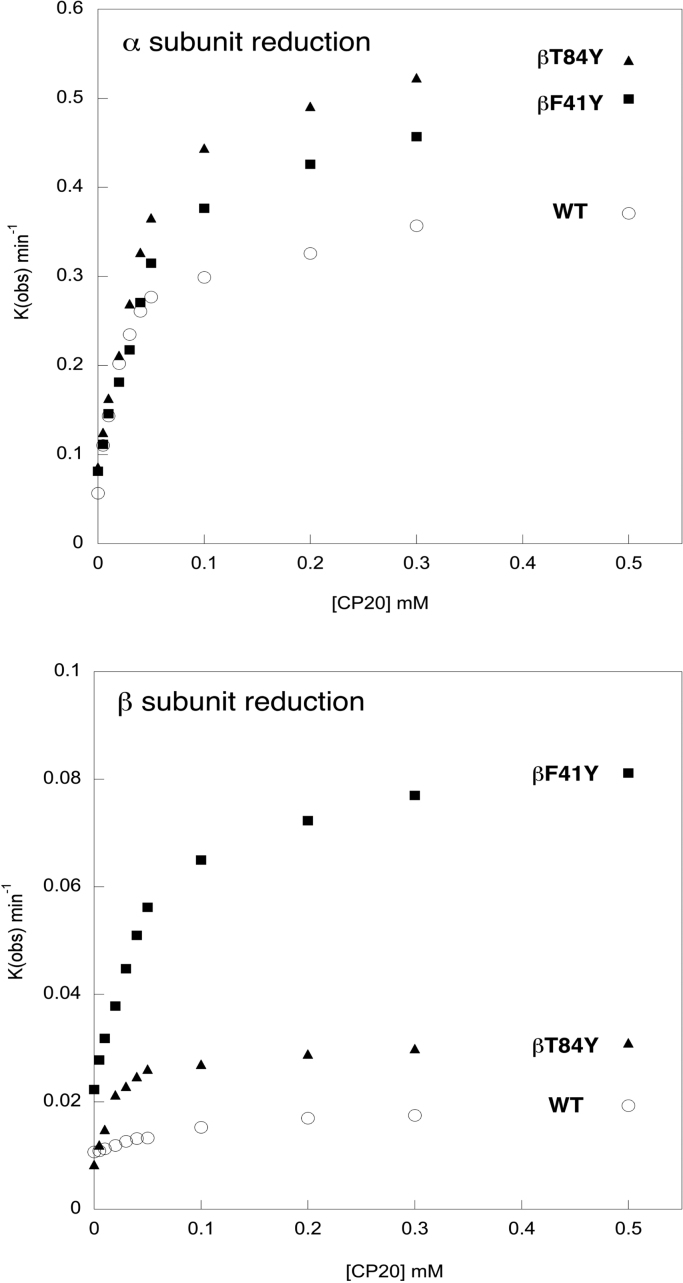
**Concentration dependence of ferryl reduction rate.** The rate constants for the ferryl reduction of selected mutants by the reductant CP20: WT (O); βF41Y (■);. βT84Y (▲). Conditions: 20 mM NaPi, pH 7.20; T = 25 °C; [heme] = 10 µM. Upper graph shows the high and low affinity α subunit rates. Lower graph shows the slower β subunit rates, and illustrates the lack of a high affinity rate in the WT β subunit lacking a surface exposed tyrosine residue.

The initial goal was to determine if it was possible to enhance the reduction rate of ferryl heme to metHb in recombinant human Hb [16] by the orders of magnitude possible to achieve in *Aplysia* Mb [17]. The hydroxypyridinone reductant CP20 has been shown to have a fast high affinity rate of electron transfer to ferryl heme in the α subunit that is saturated at low reduction concentration. In the β subunit the rate is slower and low affinity, i.e. it is either linear with or saturates only at very high reductant concentrations [16]. Fig. 2 shows the reduction of ferryl Hb as a function of the concentration of CP20 in selected mutants: WT (WT); a mutant with a tyrosine in the homologous position in the subunit as the alpha subunit (βF41Y); and a mutant with a tyrosine in an “unphysiological” position in the β subunit, but still surface exposed and close to the heme (βT84Y) All three proteins have the equivalent tyrosine in the α subunit (Y42) and all show a fast high affinity rate of reduction of the ferryl heme in the α subunit by CP20. In contrast, only the mutations with inserted tyrosine residues in the β subunit show a clearly defined high affinity saturable rate in this subunit.

Table 3 quantifies the kinetic parameters for a number of the new tyrosine mutations. In most cases inserting tyrosines into the β subunit (βF41Y, βT84Y, βL91E/βL96Y) has only small effects on the reduction of the ferryl heme in the α subunit compared to WT. Small increases in V_max_ are compensated by increases in K_m_, meaning that the calculated second order rate constant for heme reduction V_max_ /K_m_ remains largely unchanged (4–12 ×10^-3^ μM^-1^ min^-1^). Interestingly the addition of a second tyrosine near the α heme (αL91Y) had only minimal or negative effects, suggesting a limitation for heme reduction that is independent of the pathway of electrons arriving at the physiological tyrosine electron donor (αY42). In contrast, inserting a tyrosine residue close to the β heme (where none is normally present) increases both the V_max_ and lowers the K_m_ for ferryl heme reduction in this subunit. This results in a ten-fold increase in V_max_ /K_m_ (from 0.07 in WT to 0.6–1.2 ×10^-3^ μM^-1^ min^-1^ depending on the mutant). The precise position of the tyrosine seems relatively unimportant; “unphysiological” positions are at least as effective as inserting the tyrosine at the homologous site to that in the α subunit (βF41Y). Unsurprisingly, the insertion of a tyrosine near the α heme (αL91Y) did not create a high affinity route for electron transfer to the β heme. One mutant (βK66Y) showed apparently much larger rates of ferryl to met conversion in both the α and β subunits. However, on closer inspection the final spectrum formed was significantly different to the original met spectrum confirming degradative events other than simple reduction were occurring in this mutant [27].

**Table 3:** **Kinetic parameters for ferryl reduction rates of mutants.** Data taken from the conditions of Fig. 3 varying [CP20] (eleven concentrations from 5 to 1000 μM). Errors are SEM from non linear curve fitting and so not relevant for the single point value in the absence of CP20 (autoreduction rate; Auto.).

	α subunit				β subunit			
	Auto. (min ^-1^)	V_max_ (min^-1^)	K_m_ (μM)	V_max_/K_m_ (μM^-1^ min^-1^) × 10^-3^	Auto. (min ^-1^)	V_max_ (min^-1^)	K_m_ (μM)	V_max_/K_m_ (μM^-1^ min^-1^) × 10^-3^
**WT**	0.057	0.33 ± 0.01	27.9 ± 3.1	11.8 ± 1.4	0.011	0.012 ± 0.000	179.0 ± 18.1	0.067 ± 0.007
**βF41Y**	0.081	0.48 ± 0.01	67.2 ± 6.1	7.1 ± 0.7	0.022	0.069 ± 0.002	61.7 ± 6.0	1.11 ± 0.34
**βK66Y**	0.61	2.28 ± 0.06	22.7 ± 2.5	100 ± 11	0.264	1.38 ± 0.04	94.6 ± 9.3	14.6 ± 1.5
**βT84Y**	0.086	0.51 ± 0.01	48.7 ± 3.2	10.4 ± 0.7	0.008	0.024 ± 0.001	19.7 ± 1.9	1.22 ± 0.13
**αL91Y**	0.037	0.50 ± 0.01	134.6 ± 7.9	3.7 ± 0.2	0.016	0.043 ± 0.002	356.7 ± 21.6	0.12 ± 0.01
**βL91E/βL96Y**	0.016	0.72 ± 0.04	76.0 ± 13.1	9.5 ± 1.7	0.045	0.081 ± 0.008	137.0 ± 39.1	0.59 ± 0.18

**Fig. 3:**
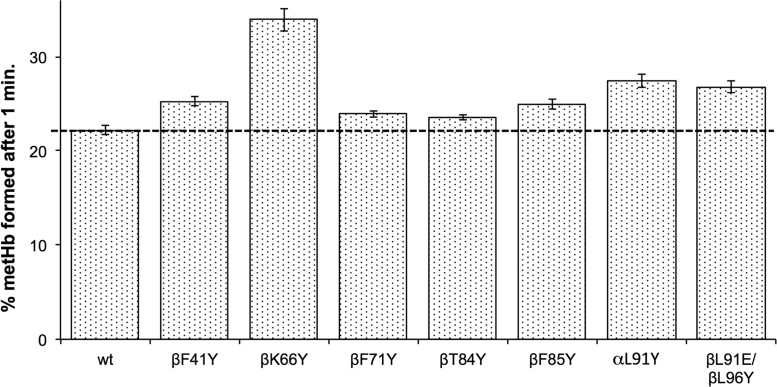
**Ascorbate reduction of ferryl heme in recombinant Hb mutants.** The % of metHb formed after one minute incubation of ferryl Hb with 30 µM ascorbate (mean ± SD, n = 3). The % metHb formed was calculated from the ∆abs.545–650 nm as a percentage of the value of ∆abs.545–630 nm for the full 100% ferryl to met conversion. Conditions: sodium phosphate (20 mM, pH 7.20); temperature = 25 °C; [heme] = 10 µM.

A comparison was then made of ferryl heme reduction using physiological levels of the plasma reductant, ascorbate (Fig. 3). After one minute all mutants with tyrosine insertions showed a significantly higher metHb formation than WT. However, neither using CP20 (Table 3) nor ascorbate (Fig. 3) do the rates of α or β ferryl heme reduction approximate the thousand-fold increase seen in some *Aplysia* Mb mutants [17].

**Fig. 4:**
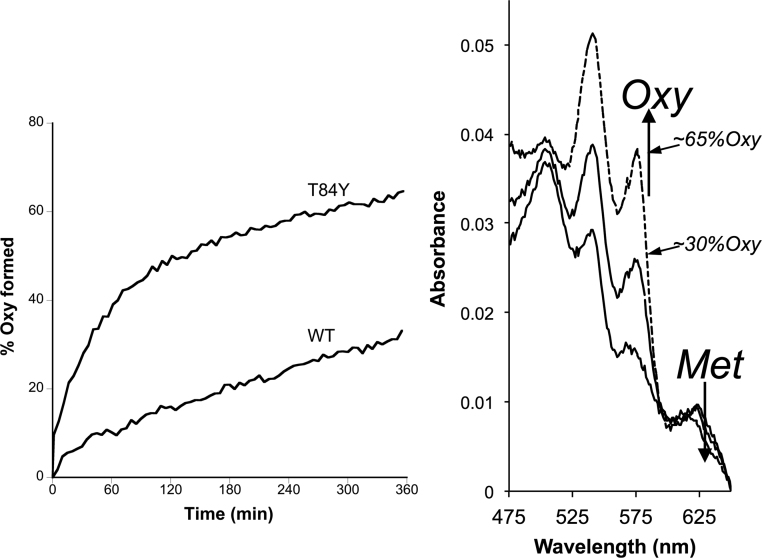
**Enhanced rate of ascorbate reduction of ferric heme in βT84Y.***Left*. Graph to show the rate of formation of oxyHb following ascorbate addition to metHb for WT and βT84Y mutant. The wavelength pair of 577–630 nm was used to monitor met to oxy formation. *Right*. Graph showing the spectra after t = 360 min (wt = long dashed line, βT84Y = short dashed line) compared to the initial t = 0 min 100% met spectrum (solid black line). Conditions: buffer = sodium phosphate (20 mM, pH 7.20); T = 25 °C; [heme] = 4.4 µM; [ascorbate] = 100 μM added at t = 0.

**Fig. 5:**
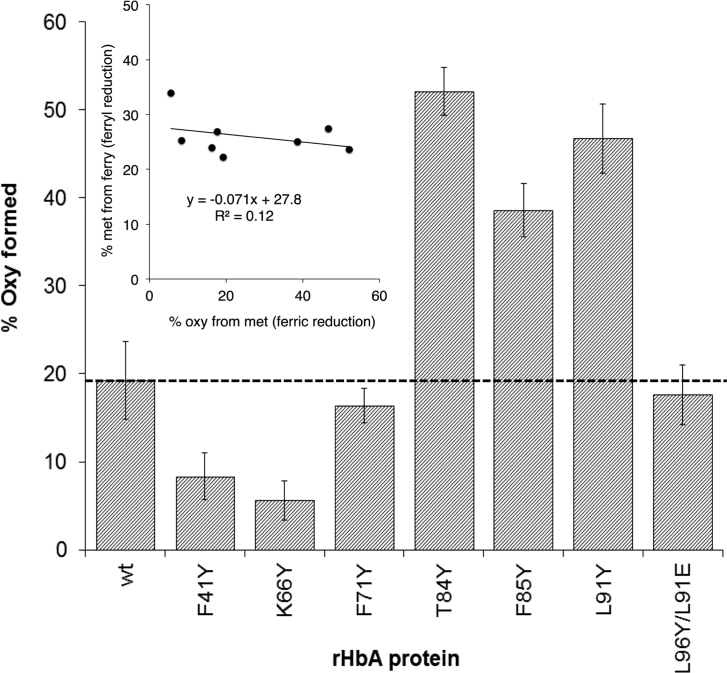
**Rate of ascorbate reduction of ferric heme in different mutants.** % oxyHb formed after 60 min incubation of following the addition of 100 μM ascorbate to 10 μM metHb (mean ± SD, n = 3). Other conditions as per Fig. 4. Inset shows lack of correlation between the rate of ferryl (Fig. 3) and ferric (Fig. 5) heme reduction in the different mutants.

Although metHb is less oxidatively damaging than ferryl Hb, it is not functional in oxygen transport and can be readily converted back to ferryl by peroxides, a process that additionally forms reactive protein-bound free radicals. It would therefore be beneficial for an extracellular HBOC to be further reduced from ferric metHb to ferrous oxyHb. In the plasma only ascorbate has the reducing power to effect this conversion. The reaction is biphasic with the faster reaction rate generally attributed to reduction of the β subunit [28]. However, at physiological levels of reductant, even in this subunit metHb conversion to oxyHb is a slow process taking hours to reach completion in native Hb. We therefore tested the ability of our mutants to accelerate met reduction to oxyHb. Fig. 4 shows a significant increase in the rate of oxyHb formation in βT84Y compared to WT. Fig. 5 compares the reduction of a variety of mutants by physiologically plausible (100 μM) levels of plasma ascorbate. WT MetHb is reduced to 20% oxyHb under these conditions. Some mutants (βT84Y; βF85Y; αL91Y) show significant increases compared to WT with over 50% metHb to oxyHb conversion whereas others (βF41Y; βK66Y) show decreases with less than 10% reduction. In a few mutants (βF71Y; βL91E/βL96Y) there is only a relatively small, if any, observable difference. Interestingly there is no correlation between how fast the ferryl Hb form of a mutant can reduced by ascorbate with how fast the metHb formed can be further reduced to oxyHb (Fig. 5, inset).

NADH and glutathione showed similar direct nonenzymatic rates of metHb reduction in WT compared to ascorbate at the same (30 μM) concentration after 60 min incubation. However, there was no increase in oxyHb formation in the βT84Y mutant (Mean %oxyHb ± SD, n = 3: glutathione WT 13.1 ± 0.9, βT84Y 9.6 ± 0.5; NADH: WT 12.4 ± 3.8, βT84Y 13.7 ± 0.5).

**Fig. 6:**
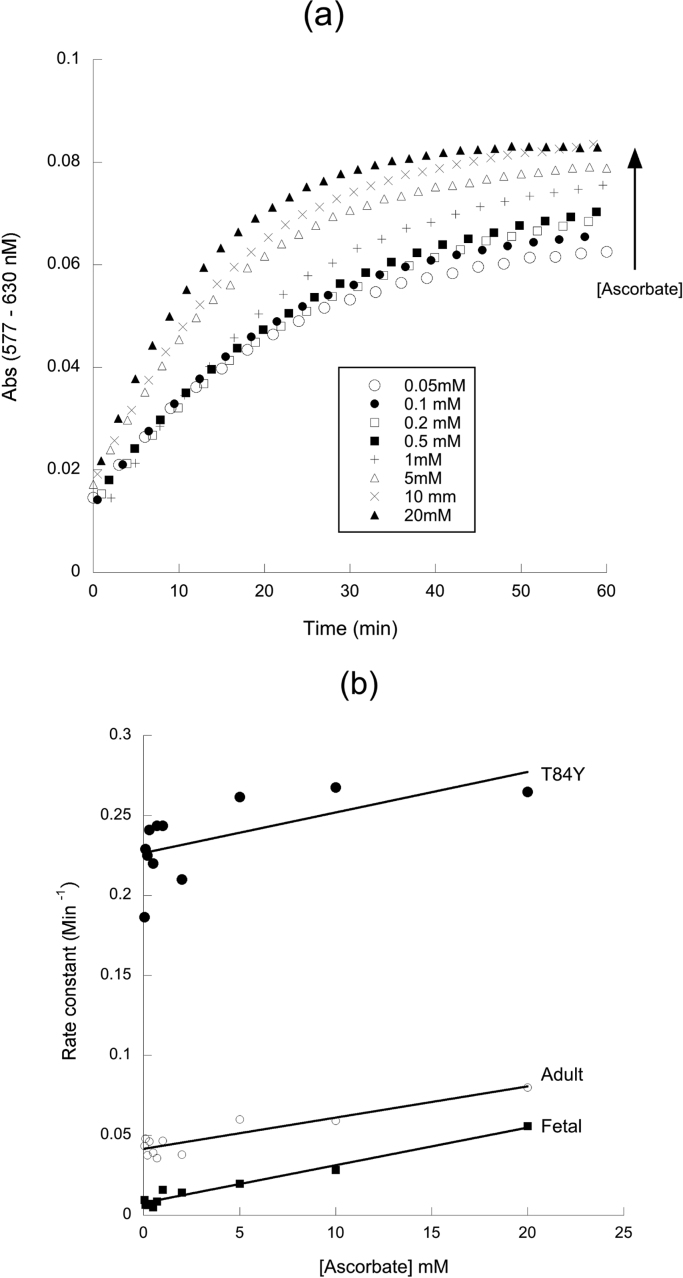
**Ascorbate concentration dependence of ferric heme reduction.** Rate of oxyb Hb formation following the addition of different concentration to metHb. (a) Time course of reduction at different ascorbate concentrations in WT Hb. (b) Pseudo first order rate constant as a function of ascorbate concentration in selected mutants. Conditions: buffer = sodium phosphate (20 mM, pH 7.20); T = 25 °C; [heme] = 8.75 µM.

A closer examination of the rate of oxyHb formation at different ascorbate concentrations (Fig. 6), reveals distinct biphasic kinetics. The faster rate has previously been attributed to preferential reduction of the β subunit [28], [29]. This rate is essentially ascorbate concentration independent (Fig. 6a) at sub mM [ascorbate]. It is this rate that differs between mutants (Fig. 6b) being most increased in the βTyr84 mutant and essentially absent in a Hb lacking the β subunit (fetal Hb). The slower – ascorbate concentration dependent – second order rate is unaffected by the addition of new tyrosine residues to the α or β subunit; it is also identical in WT adult and fetal Hb (Table 4).

**Table 4:** **Kinetic parameters for ascorbate reduction of ferric Hb mutants.** Data taken from the conditions of Fig. 5 varying ascorbate concentrations from 0.05 to 20 mM. The high affinity first order rate constant was taken from the y-axis intercept (± SEM) of a plot of the pseudo first order rate constant vs ascorbate concentration. The low affinity (second order) rate constant was calculated from the slope (± SEM) of a plot of the pseudo first order rate constant at those higher ascorbate concentrations where there was a clear ascorbate concentration dependence of the rate (1–20 mM).

	**High affinity rate** (× 10^-4^ s^-1^)	**Low affinity rate** (× 10^-2^ M^-1^ s^-1^)
**WT adult**	6.91 ± 0.33	3.16 ± 0.74
**βT84Y**	37.7^a^ ± 1.2	3.20 ± 0.23
**αL91Y**	9.28^a^ ± 0.31	3.73 ± 0.85
**βF85Y**	12.8^a^ ± 0.77	2.06 ± 0.98
**βL91E/βL96Y**	4.07^a^ ± 0.32	1.66 ± 0.40
**WT fetal**	1.27^a^ ± 0.17	3.61 ± 0.34

a Significantly different from WT adult as judged by 95% confidence intervals of curve fits.

Combining mutations in the α and β subunits that enhance ascorbate reducibility did not have an additional beneficial effect (Table 5). Crucially the most readily reducible mutant (βT84Y) did not lead to increased rates of autoxidation or heme loss from the ferric from of the protein and this mutant was therefore taken forward for further cellular and animal studies. In order to prepare a more homogenous recombinant product for cellular and animal trials, the N-terminal valine deletion was produced [22], [30], [31]. In both control and the mutant alike this led to a decrease in ascorbate reducibility, but the distinction between the two was still evident (Table 5). The V1M mutation showed a small decrease in oxygen affinity (p50 = 5.66 ± 0.26 Torr compared to WT of 5.21 ± 0.07) but had no negative effect on autoxidation or heme loss (Table 5).

**Table 5:** **Ferric reduction, heme loss and autoxidation rates of selected mutants.** Conditions: 20 mM NaPi (pH 7.2); T = 30 °C. Ferric reduction calculated as %oxy formed 60 min after 150 μM ascorbate addition to 5 μM metHb; Autoxidation rate 10 μM oxyHb; heme loss 1.7 μM metHb. Key: Unpaired *t*-test vs relevant control mutation lacking enhanced ferric reducibility.

	**Ferric reduction (n = 3)** % oxy	**Autoxidation (n = 6)** min^-1^	**Heme Loss (n = 3)** min^-1^
**WT**	51.5 ± 4.6	0.040 ± 0.010	0.028 ± 0.005
**βT84Y**	81.7 ± 2.3**	0.036 ± 0.017	0.024 ± 0.003
**αL91Y/βT84Y**	61.2 ± 1.2*	ND	ND
**αV1M βV1M**	21.4 ± 4.3	0.028 ± 0.009	0.032 ± 0.001
**αV1M βV1M βT84Y**	44.4 ± 1.4***	0.024 ± 0.008	0.035 ± 0.006

* P < 0.05 vs WT.

** P < 0.005 vs WT.

*** P < 0.005 vs αV1M βV1M.

**Fig. 7:**
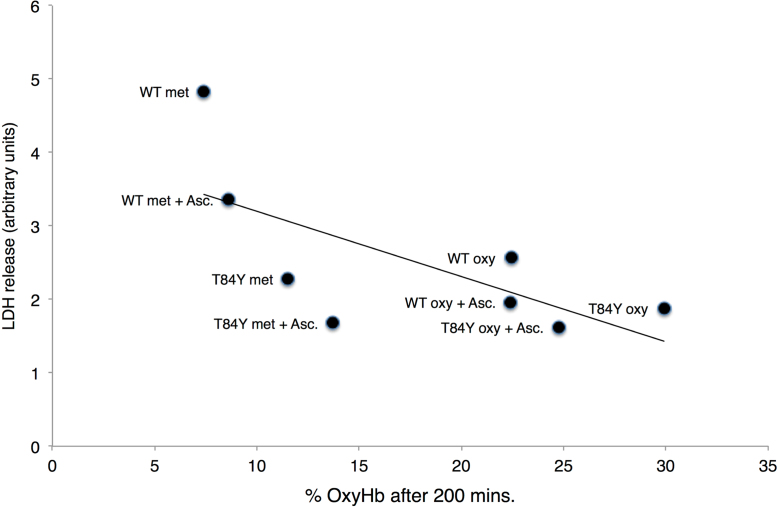
**Effect of mutations on hemoglobin-induced cell damage.** % oxyHb present 200 min after incubation of 100 μM met or oxy forms of WT and βT84Y to HEK cells in the presence or absence of 100 μM ascorbate compared to amount of LDH released 24 h. There is a significant negative correlation between the fraction of OxyHb remaining and cell damage. All Hb used also included the αV1M βV1M mutations.

The stability and oxidative reactivity of the ferrous form βT84Y was compared to WT following addition to HEK cells. Phenol red free DMEM media was used during the Hb stability and Hb-induced cell damage studies as phenol has the ability to reduce higher oxidation states of Hb and interferes with spectrophotometric assays. Hb was added to cells in both the oxy and met forms and in the presence and absence of ascorbate (Fig. 7). Following incubation with cells at 37 °C, oxyHb was converted primarily to metHb, presumably driven by autoxidation. In contrast, metHb was partially converted to oxyHb driven by the reducing power of the cell and/or the addition of 100 μM ascorbate to the media. The value of oxyHb reached a steady state after 3–4 h. In all cases the steady state oxyHb was higher in the βT84Y mutant than in WT Hb. The level of oxyHb remaining after 200 min negatively correlated with cell damage at 24 h as indicted by a decrease in the release of lactate dehydrogenase, suggesting that the ability of the βT84Y mutation to maintain higher levels of ferrous Hb was protective.

**Fig. 8:**
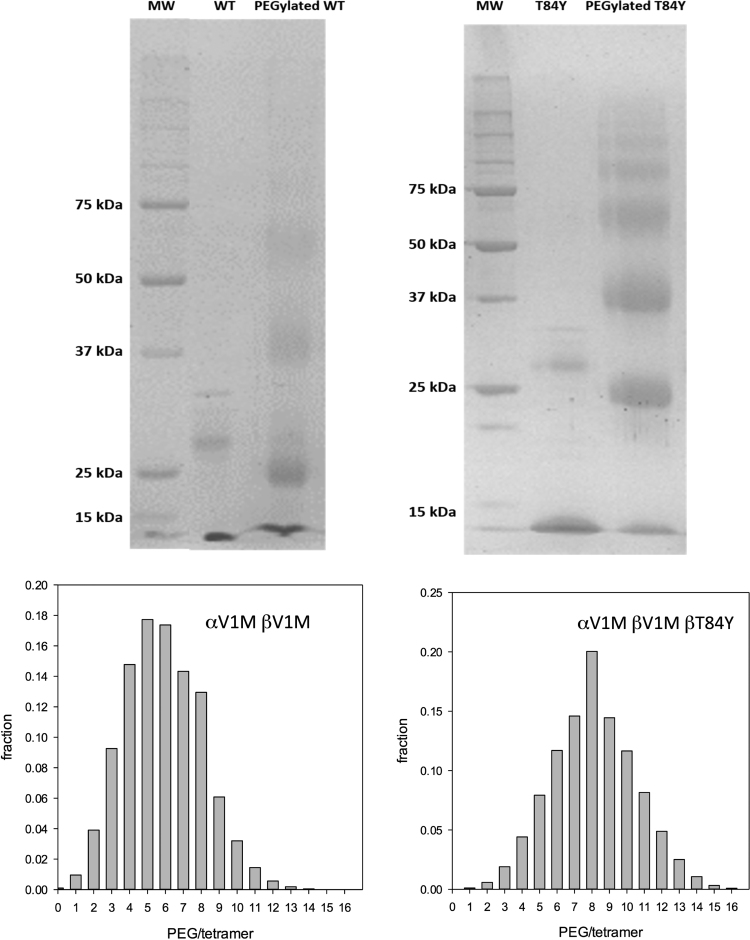
**PEGylation efficiency of mutations.** The fraction of conjugated PEG chains per tetramer was evaluated by a probabilistic calculation from the distribution of PEGylated monomers obtained by SDS PAGE.

The effect of the βT84Y mutation on in vivo vascular retention time of a HBOC was tested in a mouse top-load model. First the control (αV1M βV1M) and test (αV1M βV1M βT84Y) proteins were PEGylated in the deoxy form using the EURO-PEG-Hb method to prevent rapid renal clearance [23]. Both proteins were PEGylated at multiple sites with a slightly enhanced degree of PEGylation in the case of T84Y (Fig. 8). After PEGylation the measured oxygen affinity increased for both proteins as expected. However, for T84Y the affinity remained slightly lower (5.34 ± 0.53 Torr) than for WT (1.70 ± 0.22 Torr), consistent with what was observed for the unPEGylated mutant (Table 2).

**Fig. 9:**
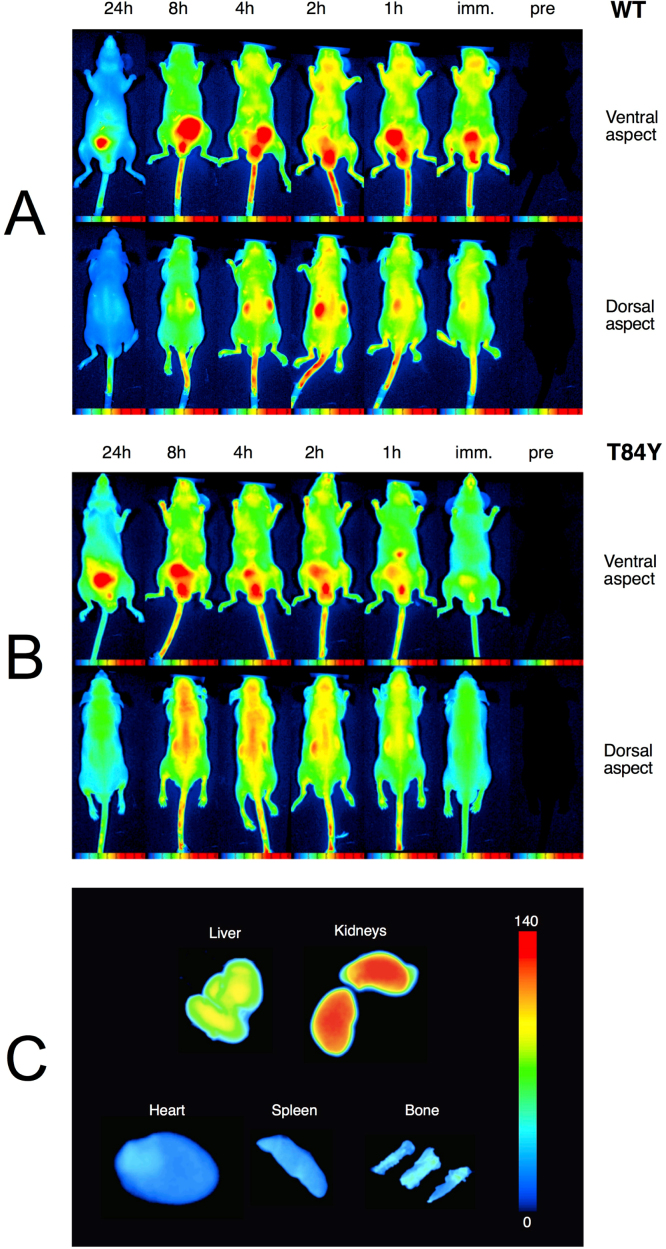
**In vivo whole-body distribution of PEGylated Hb.** Top: NIR scans shown for various time points up to 24 h following top-load infusion of WT (αV1M βV1M) and T84Y (αV1M βV1M βT84Y) PEG-Hb conjugated to the Lumiprobe Cy5. Raw intensity data are displayed in arbitrary pseudo-color scale of fluorescence intensity. Note the negligible autofluorescence of the animal (far right). Bottom: Ex vivo organ-specific direct NIR fluorescence imaging for accumulation of Cy5-labeled WT PEG-Hb.

**Fig. 10:**
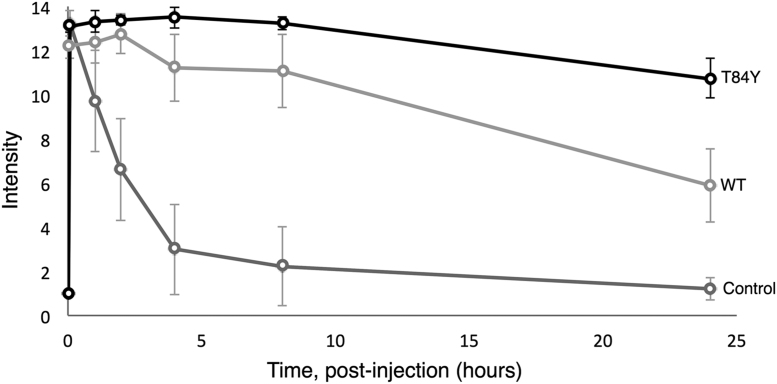
**Vascular retention and organ distribution of PEGylated Hb.** Lumiprobe Cy5 administered in saline (control) and conjugated to WT (αV1M βV1M) and T84Y (αV1M βV1M βT84Y). NIR fluorescent intensity of Cy5 in blood samples plotted as a function of time post injection.

Fig. 9 shows the whole-body distribution of the labeled proteins over a 24 h in representative animals along with their organ-level accumulation. As time progressed, the regional distribution of Cy5-Hb became heterogeneous depending on the rate of extravasation into the imaged tissues and organs (Fig. 9A and B). Note that the bladder is seen as a hot spot on these fluorescent images as all the PEG-Hb molecules passing through the kidneys accumulated here before excretion in the urine. Cases of high- and low-accumulations of labeled proteins in various organs as demonstrated by *post mortem* direct NIR imaging of the organs cleared from blood are also demonstrated (Fig. 9C). The HBOC containing the βT84Y was cleared more slowly (Fig. 9) with a significantly enhanced vascular retention time (Fig. 10).

## 4 Discussion

### 4.1 Enhanced ferryl reduction rate

We have shown previously that tyrosine residues can act as redox centres facilitating the reduction of higher oxidation states of iron in Hb [16] and in the process decreasing its oxidative reactivity [19]. Our previous mutation (βF41Y) was at the site in the β subunit, homologous to where the α subunit already possesses a redox active centre (α42). We postulated that non homologous sites located in the surface of Hb and within electron transfer range (< 10 Å) of the heme might have similar effects or even be superior to the natural and homologous sites in the relevant subunits. We also predicted that placing the mutation on the surface and distant from α/β interface should have a minimal effect on the protein expression, stability and function. From a protein design viewpoint, the intention is to have minimal effect on Hb other than in decreasing oxidative reactivity. This would enable the mutation to be readily incorporated in a HBOC that could then have additional mutations to enhance stability and/or affect other functions, such as decreasing nitric oxide scavenging [5]. With one exception this goal was achieved. The exception was the βK66Y mutation, which could be readily expressed and purified but was unstable and heterogenous in the oxidized ferric and ferryl states. So although this mutant had the fastest ferryl reduction rates, in part this was due to enhanced autoreduction to ferric forms that could not be recovered to functional Hb. This mutation was therefore deemed unstable as a component of a HBOC and not studied further.

All the other six new mutations in the β subunit showed evidence of a new redox active centre that could increase ferryl reduction by exogenous reductants. There was little effect on reduction at the unaltered α site, confirming the specificity of the mutations to the subunit in which they resided. The introduction of a second (non physiological) tyrosine redox centre in the α subunit (αL91Y) did not seem to greatly enhance reduction at this site, although there was a modest increase in the overall rate, possibly via the creation of a new pathway to the β heme.

In general, although the design decisions were vindicated by the introduction of these new redox pathways, the overall increase in reduction rate was somewhat disappointing. None of the mutations showed reduction of the β subunit as fast as that present naturally in the WT α subunit. The engineered proteins were reduced orders of magnitude slower than that observed following the introduction of tyrosine redox centres into the *Aplysia* Mb model system [17], despite the fact that a number of the mutations were engineered to be as homologous as possible to the relevant *Aplysia* sites (Table 1). All the natural and engineered mutations are close enough to the heme group (< 10 Å) such that according to Marcus theory the rate of electron transfer should not be limited by the nature of the intervening protein medium [32]. Indeed the fact that introducing tyrosine residues at multiple sites leads to broadly similar increases in electron transfer rate confirms that the chemical nature of the electron transfer pathway from tyrosine to heme is not important.

If the reduced tyrosine radical is formed at 100% occupancy by the addition of saturating levels of reductant the V_max_ value in Table 3 should be on the millisecond, rather the minutes, time scale. We have previously postulated that the reason for the rather slow electron transfer rates of ferryl reduction from nearby tyrosine residues was due to the relevant reactive species only being present in low population [15]. Thus, the fast intrinsic electron transfer rate (≈ 10^8^ s^-1^) would have to be multiplied by a low electron occupancy of the relevant reactive reduced species to generate the effective rate, the deprotonated tyrosine radical and the protonated ferryl being the relevant reactive species. It is possible that the faster rate in *Aplysia* is therefore due to a higher intrinsic pK of the protonated ferryl, resulting in a larger population of the reactive species. A limitation due to the intrinsic nature of the ferryl heme in different species/proteins would also explain why changing the position of the redox active tyrosine in Hb has only a small effect on the electron transfer rate. However, as even this relatively modest increase is accompanied by a decrease in lipid peroxidation in cell free assays [19] the addition of such tyrosine residues would still be a useful component to an engineered HBOC.

### 4.2 Enhanced ferric reduction rate

Given the high oxidation potential [12], [33] of the ferric/ferryl species (≈ + 1 V), many mild reductants, both unphysiological such as the hydroxypyridonones [16], [34] and physiological such as urate [9], [35] have the thermodynamic power to readily reduce ferryl to ferric Hb. However, given the redox potential of ferric/ferrous Hb (≈ -50 to 100 mV), stronger reductants are required to further reduce the ferric species to ferrous. In plasma where HBOC are designed to function, this is therefore likely limited to glutathione, NADH and ascorbate. The ascorbate reduction rate is non trivial. Indeed as noted previously [16], the further conversion of ferric to ferrous Hb by ascorbate can complicate the analysis of the reduction kinetics of ferryl Hb by ascorbate. It was therefore relevant to test whether the insertion of novel tyrosine mutations that enhance the conversion of ferryl to ferric Hb, also enhanced the conversion of ferric to ferrous Hb.

The ability of ascorbate to reduce directly human met to oxyHb was first shown in the 1940s [36], [37]; this rate was later calculated to be in the order of 1 M^-1^ s^-1^ and enhanced by the addition of organic phosphates [38], [39]. MetMb is also reduced by ascorbate, but a slower second order rate constant of between 10^-3^ and 10^-2^ M^-1^ s^-1^ [40]. Glutathione and NADH reduction of metHb is likely to occur at a slower rate than ascorbate in vivo *–* this paper and [10], [36], [41] – especially given that plasma levels of ascorbate in humans (≈ 50 μM) are significantly higher than those of NADH [42] and GSH [43].

A more detailed examination of the ascorbate metHb reduction rate showed that the reaction proceeded in two stages, with the reduction of the β being significantly faster than that of the α subunit [28], [29], the latter likely being more akin to that seen in Mb. Consistent with this fetal Hb, although having a similar reduction potential to adult Hb [44], lacks a β subunit and has slower reduction kinetics than the adult protein [20].

The results in this paper significantly enhance our knowledge of the mechanism of ascorbate reduction of metHb. Fig. 6b shows that in WT Hb, as ascorbate concentration is varied the reaction obeys second order kinetics. However, there is a significant positive intercept on the y-axis. The difficulty of making measurements under pseudo first order conditions at very low ascorbate concentrations precluded us from a more detailed analysis. However, we do not feel that this indicates reduction occurring at very low or zero ascorbate concentrations and instead favor an explanation akin to what occurs with ascorbate reduction of ferryl Hb. In classical chemical kinetics, a non zero y-axis intercept could indicate the rate of the reverse reaction, but the ascorbate reduction of metHb is not readily reversible, especially in the presence of oxygen which will drag the reaction in the direction of the ferrous oxy state. Instead the lack of concentration dependence at low ascorbate (Fig. 6b) likely indicates the saturation of a high affinity (low μM or less) binding site similar to that seen for the reduction of ferryl Hb [16]. This is consistent with the original finding by Gibson that ascorbate reduction at low concentrations is much faster than expected by second order kinetics [36]. Similar non linear reduction kinetics at lower ascorbate concentrations was also later shown by McGown [45]. Although Gibson attributed the zero order kinetics at low ascorbate to trace metal contamination in solution, a similar effect in the Hb peroxidase kinetics was confirmed by NMR to be due to a specific ascorbate binding site [46]. The effect of this high affinity binding site is to make ascorbate reduction of the Hb β subunit at physiological (μM) plasma ascorbate concentrations as fast as those globins, such as cytoglobin, which have much faster reduction rates than Hb at higher (mM) ascorbate [47].

Although the ferric reduction kinetics by ascorbate are similar to the ferryl reduction kinetics in having a specific high affinity saturable component and a non specific low affinity component, the underpinning mechanisms are different. This is why there is no correlation between those mutants that enhance ferryl reducibility and those that enhance ferric reducibility (Fig. 5). Table 4 shows that there is no effect of inserting tyrosine residues on the low affinity rate. Nor is this rate different in adult and fetal Hb. The calculated range of low affinity rates (1–4 × 10^-2^ M^-1^ s^-1^) is consistent with that previously reported for metMb and therefore likely relates to the direct second order reduction of the heme by ascorbate. A more positive heme redox potential was postulated to underpin an enhanced reducibility of the ferric heme in *Lumbricus* Hb [41]. The similarity of the direct low affinity ascorbate reduction rates for WT Hb, fetal Hb and our adult Hb mutants suggest that this effect is not present here.

The faster rates at low ascorbate concentrations are likely caused by the specific ascorbate binding site on the β subunit. The addition of tyrosine residues that decrease this rate (βF41Y, βK66Y, βF71Y, βL91E/βL96Y) might act via minor structural changes to the protein perturbing the ascorbate binding site. Mutations that increase this rate (βT84Y, αL91Y, βF85Y) might also act on the binding site, but it is equally possible that they enable an additional electron transfer pathway from the ascorbate binding site to the β or even the α heme. This seems especially likely for the α subunit mutation (αL91Y) and the β mutation that has the fastest rate of electron transfer (βT84Y).

### 4.3 In vivo studies

We have previously demonstrated that engineered human HBOC with an increased reducibility of ferryl Hb by physiological plasma reductants decrease Hb-induced oxidative stress [19]. The increased reducibility of ferric to ferrous Hb found in the multimeric Hb (erythrocruorin) in earthworms (*Lumbricus*) has also been promoted as a benefit of HBOC [41]. βT84Y has both these positive features, is no worse than WT Hb in terms of autoxidation and heme loss (Table 5) and, in the presence of ascorbate, maintained Hb in the oxy state decreasing cell damage (Fig. 7). We therefore chose it as a component of a HBOC that would decrease oxidative stress and remain longer in the functional ferrous state in vivo. Converting Hb into a HBOC requires increasing the effective size of the dimer to prevent premature renal clearance via cross-linking, polymerization or conjugation. We chose to PEGylate βT84Y in the deoxy state using the EURO-PEG-Hb method as this has been shown to have advantages over PEGylation in the oxy state, such as decreased tendency to dimerization [48]. PEGylation was successful; the oxygen affinity of PEGylated βT84Y (≈ 5 Torr) was similar to that in the Sangart HBOC product, MP4, proposed to facilitate oxygen transfer from the red blood cell to hypoxic tissues [11].

PEG-Hb labeling studies showed the PEGylated proteins had a plasma half time of several days (Figs. 9 and 10), in agreement with our own [23] and other such products that have been tested [49] and ideal for an oxygen therapeutic product used in acute clinical settings. There have been relatively few studies on tissue uptake of PEG-Hb and none in a mouse model. In a rat model Bovine PEG-Hb showed accumulation in the liver and kidneys and an intravascular half-life of 17–19 h comparable to our findings shown in Figs. 9 and 10 [50], [51]. In a mouse model [52] the uptake of PEGylated nanoparticles containing Hb also found high retention in liver and kidneys and low retention in heart, spleen and bone, again consistent with our data.

The βT84Y product was more PEGylated (Fig. 8) than WT and had an enhanced vascular retention time (Figs. 9 and 10). PEGylation differences may be partly responsible for the enhanced retention. However, the βT84Y product is more readily reducible. Heme loss is enhanced from the ferric state of Hb [18] and a protein with no heme is likely to be cleared more quickly than ferrous Hb. Increased vascular retention time can also be due to a decrease in oxidative stress caused by the T84Y mutant [53] as oxidative stress could increase vascular escape via a damaged endothelial layer [54]. Alternatively a direct explanation is possible if the surface charge of the protein is modified as permeability is decreased the more negative the surface charge [23], [55] Future studies will be needed to determine if this vascular enhancement was a function of the more effective PEGylation, modification to the protein surface charge, enhanced ascorbate reducibility or and/or decreased oxidative stress.

## 5 Conclusion

Ascorbate has been shown to be the key plasma antioxidant for HBOC, maintaining it in the ferrous state and reducing oxidative species such as ferryl and protein free radicals [9], [10], [56]. Key to this is the ability of the erythrocyte transmembrane reductase system to use intracellular ascorbate as a reductant for the oxidized ascorbate radical in plasma, maintaining levels of the reduced species [10], [45], [57], [58]. When these are compromised in vivo, for example in species such as humans and guinea pigs that lack the ability to synthesis ascorbate *de novo*, HBOC cause increased oxidative stress [9], [56]. The results in this paper show that Hb has a high affinity ascorbate binding site such that the rate of both ferryl and ferric reduction is not significantly compromised until ascorbate levels fall significantly below normal plasma levels. It is also possible to engineer mutations to enhance the reducibility of Hb in a HBOC. Mutations that incorporate these features, such as βT84Y, may therefore be useful as components of a less oxidatively damaging HBOC, which has increased vascular retention. Knowledge of the position and structure of the ascorbate binding site in the β subunit will possibly enable future mutants with engineered electron transfer pathways from this site to the α subunit heme or, more speculatively, incorporate an additional binding site into the adult α and/or fetal γ subunits.

## Funding

We would like to thank the Biotechnology and Biological Sciences Research Council, United Kingdom for financial support (BB/L004232/1).

## Disclosures

CEC, BJR and GGAS have patents granted and pending relating to modification of hemoglobin amino acids designed to render a blood substitute less toxic and are shareholders in a related company (CymBlood).
