# Supplementary material for: Engineering tyrosine residues into hemoglobin enhances heme reduction, decreases oxidative stress and increases vascular retention of a hemoglobin based blood substitute
Source: Free Radic Biol Med. 2019 Apr;134:106–18. doi: 10.1016/j.freeradbiomed.2018.12.030 (PMC6597946; doi:10.1016/j.freeradbiomed.2018.12.030)
Supplement: Supplementary file 1 — Supplementary material [file mmc1.pdf]

SUPPLEMENTARY MATERIAL (Figure S1)

To characterize the amount of free Cy5 remaining in the Cy5-labeled HBOC reaction mixture, fractionated collection of both the free Cy5 dye solution and the Cy5-labeled HBOC solution was run through the NAP-10 SEC column using a Sephadex G25-filled column in Tricorn 5/50, with phosphate buffered saline as eluent, and a flow rate of 0.25 ml/min. A JASCO HPLC system was used with a UV detector at 254 nm. During elution, 0.5 mL fractions were taken. In **Fig. S1**, the top panel fractions are of the free Cy5 dye while those in the middle panel show the combined distribution of the earlier eluting Cy5-labeled HBOC (dark green/red) and the later eluting free Cy5 molecules (blue). It is clearly seen that the free dye (blue) was effectively removed from the Cy5-labeled HBOC reaction mixture using NAP 10 (bottom panel). The double peak in the purified eluent's spectrum is due to layer-wise inhomodeneity in the SEC column as was demonstrated by a control run of a liposomal mixture (not shown).

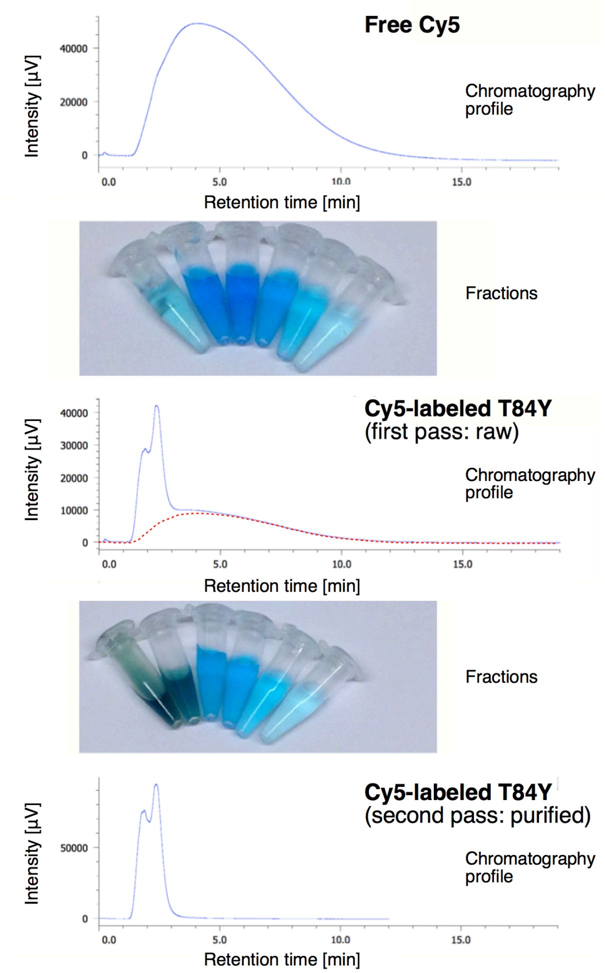

Fig. S1 Quality controlling the Cy5-lebeling of HBOC molecules

Solution of Lumiprobe Cy5-labeled HBOC (T84Y in this example) run through the column twice (i.e. in a two-pass process) did not contain free NIR Cy5 dye (bottom panel). Spectra and associated fractions are seen in the upper two panels. Dotted red curve in the middle panel is a downscaled replica of the free Cy5 curve (top panel) demonstrating that the first-pass Cy5-labeled HBOC curve (blue curve in the middle panel) is still a mixture of HBOC-bound and free Cy5. In the second-pass (bottom panel) however, the free Cy5 was effectively removed yielding a purified Cy5-labeled HBOC solution suitable for *in vivo* application for intravascular levels and biodistribution patterns.
